# *Gynostemma pentaphyllum* saponins attenuate inflammation *in vitro* and *in vivo* by inhibition of NF-κB and STAT3 signaling

**DOI:** 10.18632/oncotarget.20997

**Published:** 2017-09-18

**Authors:** Wing-Yan Wong, Magnolia Muk-Lan Lee, Brandon Dow Chan, Victor Wan-San Ma, Wenchun Zhang, Timothy Tak-Chun Yip, Wing-Tak Wong, William Chi-Shing Tai

**Affiliations:** ^1^ Department of Applied Biology and Chemical Technology, The Hong Kong Polytechnic University, Hong Kong S.A.R., China; ^2^ State Key Laboratory of Chinese Medicine and Molecular Pharmacology (Incubation), Shenzhen Research Institute of The Hong Kong Polytechnic University, Shenzhen, China; ^3^ Department of Clinical Oncology, Queen Elizabeth Hospital, Hong Kong S.A.R., China

**Keywords:** gynostemma pentaphyllum saponins, inflammatory bowel disease, macrophages, colitis, anti-inflammation

## Abstract

Recent advances in the development of anti-inflammatory agents have improved their therapeutic outcome in inflammatory bowel disease (IBD), however, the presence of side effects and limited effectiveness hinder their widespread use. Therefore, novel compounds with strong anti-inflammatory efficacy are still required. In this study, we investigated the anti-inflammatory effect and potential mechanisms of *Gynostemma pentaphyllum* (Thunb.) Makino saponins (GpS), a major component of the herbal medicine widely used in Asian countries. In *in vitro* studies, we demonstrated that GpS dose dependently suppressed activation of macrophages, one of the main effectors in IBD. GpS also suppressed cytokine production and the activation of NF-κB and STAT3 signaling in lipopolysaccharide-induced macrophages, without affecting their viability. Further *in vivo* studies demonstrated that GpS could ameliorate the weight loss, increased disease activity index, colon shortening and histological damage associated with dextran sulfate sodium (DSS)-induced colitis in mice. In agreement with results from our *in vitro* experiments, GpS suppressed cytokine production and activation of NF-κB and STAT3 signaling in the colons of DSS-induced mice.

In this study, we present for the first time, evidence of the therapeutic effect of GpS in IBD, highlighting its potential as an effective therapeutic against the disease.

## INTRODUCTION

Inflammatory bowel disease (IBD) is a chronic, relapsing gastrointestinal inflammatory disorder comprising as its two major forms, ulcerative colitis (UC) and Crohn's disease (CD). It is estimated that over 3.7 million people worldwide suffer from IBD, and incidence and prevalence rates continue to increase. North America and Western Europe are traditionally regarded as high risk areas for IBD, however, emerging populations such as those in Asia have exhibited rapid increases in the prevalence of IBD over the past several decades. Although the exact etiology of IBD requires further study, interactions between causal factors for the disease - environment, host genetics, immune system and microbiome - are known to be important [1, 2]. In particular, a dysregulated immune system has been acknowledged to be a major contributor to the pathogenesis and progression of IBD [3, 4].

In the pathogenesis of IBD, intestinal immune homeostasis is disturbed, resulting in uncontrolled activation of innate and adaptive immune cells. Macrophages are one of the most abundant innate immune cell types in the intestine and play a key role in the pathogenesis of IBD [5, 6]. In the active state of the disease, macrophages in the intestine are activated and respond by releasing proinflammatory cytokines such as inducible nitric oxide synthase (iNOS), cyclooxygenase-2 (COX-2), interleukin (IL)-6, IL-12 and tumor necrosis factor-alpha (TNF-α). The increased production of proinflammatory molecules attracts more inflammatory cells to the intestine, amplifying and extending the inflammatory response. Tissue damage ultimately results from the release of noxious mediators [7–9].

As one of the major regulators of inflammation, NF-κB plays a pivotal role in the activation of macrophages. In the inactive state, NF-κB associates with IκB and is retained as a complex in the cytoplasm. Upon activation, IκB is phosphorylated by the IKK-α/β complex and then recognized by ubiquitinating enzymes for proteasomal degradation. The dissociated NF-κB will translocate into the nucleus and activate transcription of proinflammatory genes [10, 11]. Emerging evidence also suggests a role for STAT3 in mediating the activation of macrophages. It has been shown that STAT3 is frequently phosphorylated in activated macrophages and is responsible for transcriptional induction of proinflammatory mediators [12]. Importantly, both NF-κB and STAT3 are implicated in the pathophysiology of IBD, as well as the development of colorectal cancer (CRC). It has been shown that NF-κB and STAT3 are markedly induced in the intestines of IBD patients and strongly influence the inflammatory process [13, 14]. Sustained inflammatory responses in the intestines of IBD patients increase their risk for CRC, proportional to the severity, extent and duration of the inflammation [15]. Therefore, suppression of activated NF-κB and STAT3 is a major focus of IBD treatment.

In recent years, herbal medicines have been widely used amongst IBD patients as complementary and alternative medicines [16, 17]. Herbal medicines can not only improve the condition of IBD patients, but are also efficient in suppressing activated NF-κB and STAT3, indicating their potential as a source for the discovery of anti-inflammatory agents and therapeutics for IBD [18–21]. *Gynostemma pentaphyllum* (Thunb.) Makino (Gp) has been widely used as an herbal medicine in Asian countries. Saponins (GpS) are one of the major active components in Gp and to date there have been more than 100 dammarane-type GpS identified in Gp [22–24]. Previous studies have demonstrated that long-term treatment with Gp does not induce *in vivo* toxicity, and accumulating evidence has suggested the beneficial effects of GpS in a wide range of chronic diseases [25]. In particular, the strong reactive oxidative species (ROS) scavenging activity of GpS is thought to be important for its activity [26–29]. As ROS may initiate inflammation via activation of NF-κB, GpS may potentially exhibit anti-inflammatory effects through suppression of NF-κB [30]. Previous studies have demonstrated the NF-κB-inhibitory effect of GpS in activated macrophages and our group has also shown that GpS is an effective inhibitor of STAT3 in the intestinal epithelium and polyps of CRC *Apc^Min/+^* mice [31, 32]. Given the importance of NF-κB and STAT3 in the pathogenesis of IBD, we hypothesized that GpS may potentially be effective against IBD.

In this study, we aimed to investigate the anti-inflammatory effect of GpS and to elucidate the potential mechanisms involved. Using the lipopolysaccharide (LPS)-induced murine macrophage model and the dextran sulfate sodium (DSS)-induced acute colitis mouse model, we have demonstrated the potent anti-inflammatory effect of GpS. GpS could suppress the inflammatory response via inhibition of NF-κB and STAT3 signaling *in vitro* and *in vivo*, and could also ameliorate the manifestation of acute colitis in DSS-induced mice. Our findings present strong evidence of the anti-inflammatory effects of GpS and highlight its potential as a therapeutic agent for the treatment of IBD.

## RESULTS

### GpS did not affect the cell viability of RAW264.7 macrophages and suppressed NO production and iNOS expression in LPS-induced RAW264.7 macrophages

**Figure 1:**
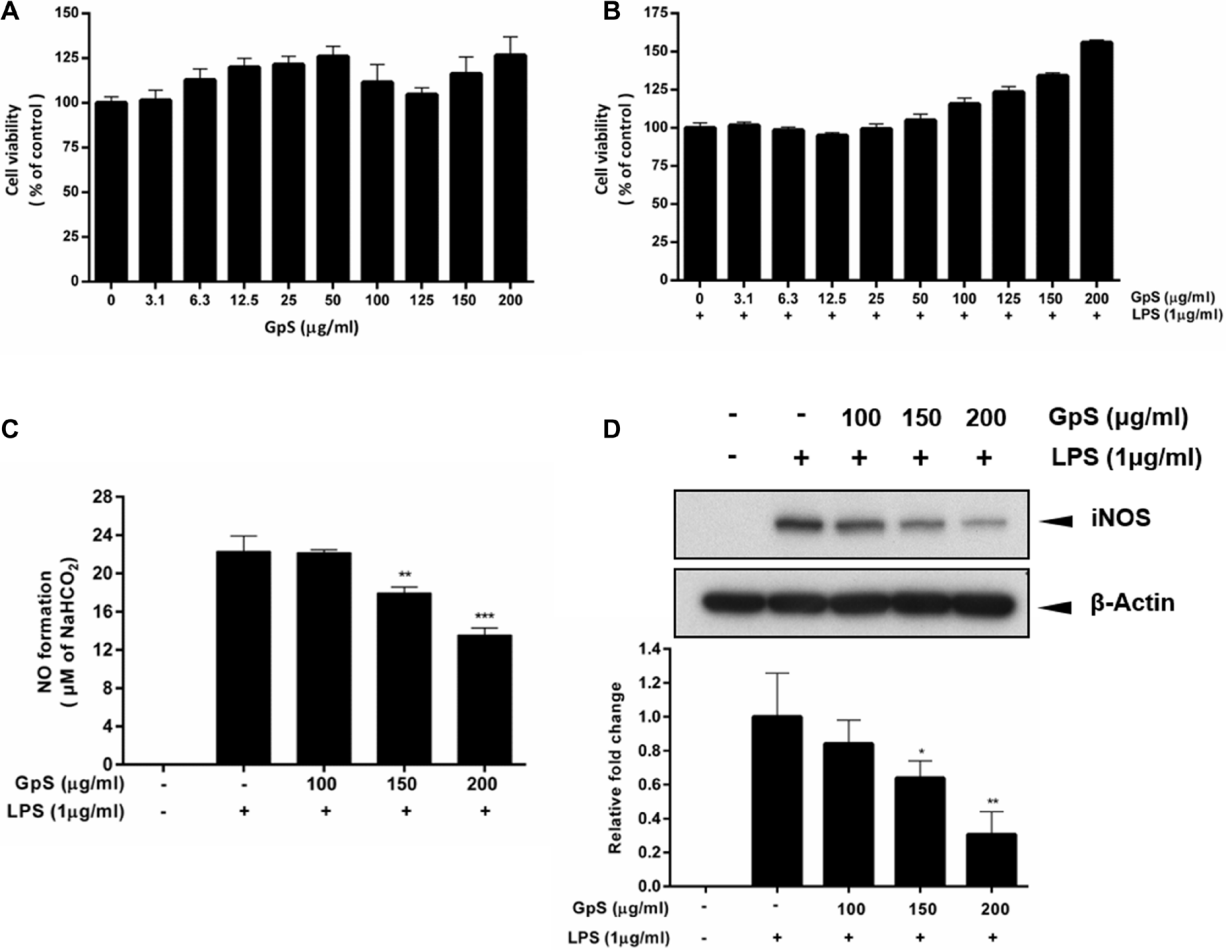
GpS inhibits LPS-induced NO production and iNOS expression without affecting the cell viability of RAW 264.7 macrophages Cells were treated with GpS at concentrations ranging from 3.1 to 200 μg/ml in the (**A**) absence or (**B**) presence of LPS (1 μg/ml) for 24 hrs. Cell viability was determined by MTT assay. Cells were pre-treated for 1 hr with indicated the concentrations of GpS and further incubated for 24 hrs with or without LPS (1 μg/ml) before assement of (**C**) NO production and (**D**) iNOS protein expression by Griess assay and Western blotting respectively. Representative immunoblot results and their quantifications are shown. β-actin was used as an internal loading control. Data are expressed as means ± SD of three independent experiments. ^*^P < 0.05, ^**^P < 0.01, ^***^P < 0.001 compared to LPS-induced control.

To examine the cytotoxicity of GpS in RAW264.7 macrophages, we treated the cells with 3.1 to 200 μg/ml GpS for 24 hrs and observed its effect on cell viability. Our results showed that there was no significant difference in the viability of cells treated with GpS compared to control, suggesting GpS did not exhibit cytotoxicity in RAW264.7 macrophages (Figure 1A). We also examined the effect of GpS in macrophages stimulated by LPS. In line with the results from unstimulated macrophages, GpS did not affect the cell viability of activated macrophages (Figure 1B) and therefore, doses of 100, 150 and 200 μg/ml were selected for downstream experiments. Next, we investigated the effect of GpS on NO production in LPS-induced macrophages. LPS-induced NO production in macrophages was significantly decreased by treatment with GpS, in a dose-dependent manner (Figure 1C). We further examined the effect of GpS on the expression of iNOS, the upstream regulator for the production of NO. As shown in Figure 1D, GpS significantly suppressed the protein expression of iNOS. Notably, when compared with vehicle control, 200 μg/ml GpS significantly suppressed the LPS-induced expression of iNOS by about 70%. These results suggested that in LPS-induced macrophages, GpS could inhibit NO production and iNOS expression, without affecting cell viability.

### GpS suppressed NF-κB signaling in LPS-induced RAW264.7 macrophages

**Figure 2:**
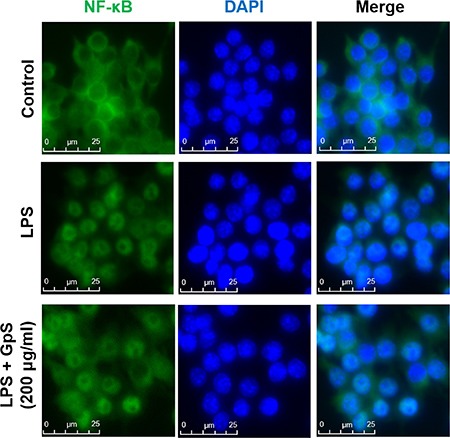
GpS inhibits nuclear translocation of NF-κB/p65 in LPS-induced RAW 264.7 macrophages Cells were pre-treated with or without GpS (200 μg/ml) for 12 hrs and then exposed to LPS (1 μg/ml) for 1 hr. Cells were then fixed, permeabilized and processed for immunofluorescent staining of NF-κB/p65. Nuclei were stained with DAPI.

**Figure 3:**
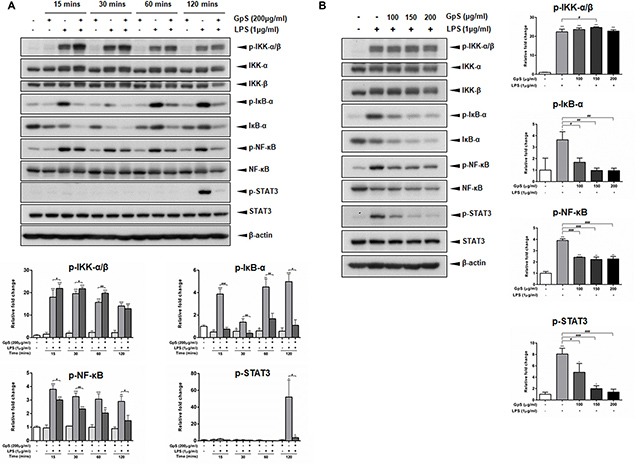
GpS inhibits IκB-α, NF-κB and STAT3 phosphorylation in LPS-induced RAW 264.7 macrophages (**A**) Cells were pre-treated with or without 200 μg/ml GpS for 1 hr and then exposed to LPS (1 μg/ml) for the indicated times. (**B**) Cells were pre-treated with or without different concentrations of GpS for 1 hr and then exposed to LPS (1 μg/ml) for 2 hrs. The protein levels of phospho-IKK-α/β, IKK-α, IKK-β, phospho-IκB-α, IκB-α, phospho-NF-κB, NF-κB, phospho-STAT3 and STAT3 were detected by Western blotting. β-actin was used as an internal loading control. Three independent experiments were performed and representative immunoblots and quantifications are shown. Data are expressed as means ± SD; ^*^P < 0.05, ^**^P < 0.01, ^***^P < 0.001 compared to untreated control; ^#^*P* < 0.05, ^##^*P* < 0.01, ^###^*P* < 0.001 compared to LPS-induced control.

Upon stimulation by inflammatory signals, NF-κB signaling is activated, leading to translocation of NF-κB/p65 into the nucleus of the cell and the consequent expression of multiple inflammatory and innate immune genes [10, 11]. Therefore, we first investigated the effect of GpS on the nuclear translocation of NF-κB/p65 in LPS-induced RAW264.7 macrophages. As shown in Figure 2, when compared with vehicle control, GpS treatment suppressed translocation of NF-κB/p65 into the nucleus of LPS-induced macrophages. Further, we examined the effect of GpS on the expression of phosphorylated NF-κB and its upstream regulators, IκB-α and IKK-α/β by Western blotting. In comparison with vehicle control, 200 μg/ml GpS significantly downregulated the expression of phosphorylated NF-κB and IκB-α in LPS-induced macrophages in a time dependent manner (Figure 3A). We then studied the dose dependent effect of GpS on LPS-induced macrophages. Our results showed that GpS treatment significantly and dose dependently suppressed the expression of phosphorylated NF-κB and IκB-α (Figure 3B). Taken together, these results suggested that GpS could suppress the activation of NF-κB signaling in LPS-induced macrophages, indicating its potential anti-inflammatory activity.

### GpS suppressed STAT3 signaling in LPS-induced RAW264.7 macrophages

Recently, STAT3 signaling has been shown to be a major contributor to the induction and maintenance of inflammation [12]. Therefore, we sought to examine if GpS could also mediate STAT3 signaling in LPS-induced macrophages. As shown in Figure 3, in LPS-induced macrophages, when compared with the vehicle control, GpS treatment significantly suppressed the expression of STAT3 in a time and dose dependent manner, suggesting that GpS could also inhibit STAT3 signaling during inflammation.

### GpS ameliorated the severity of DSS-induced acute colitis in mice

**Figure 4:**
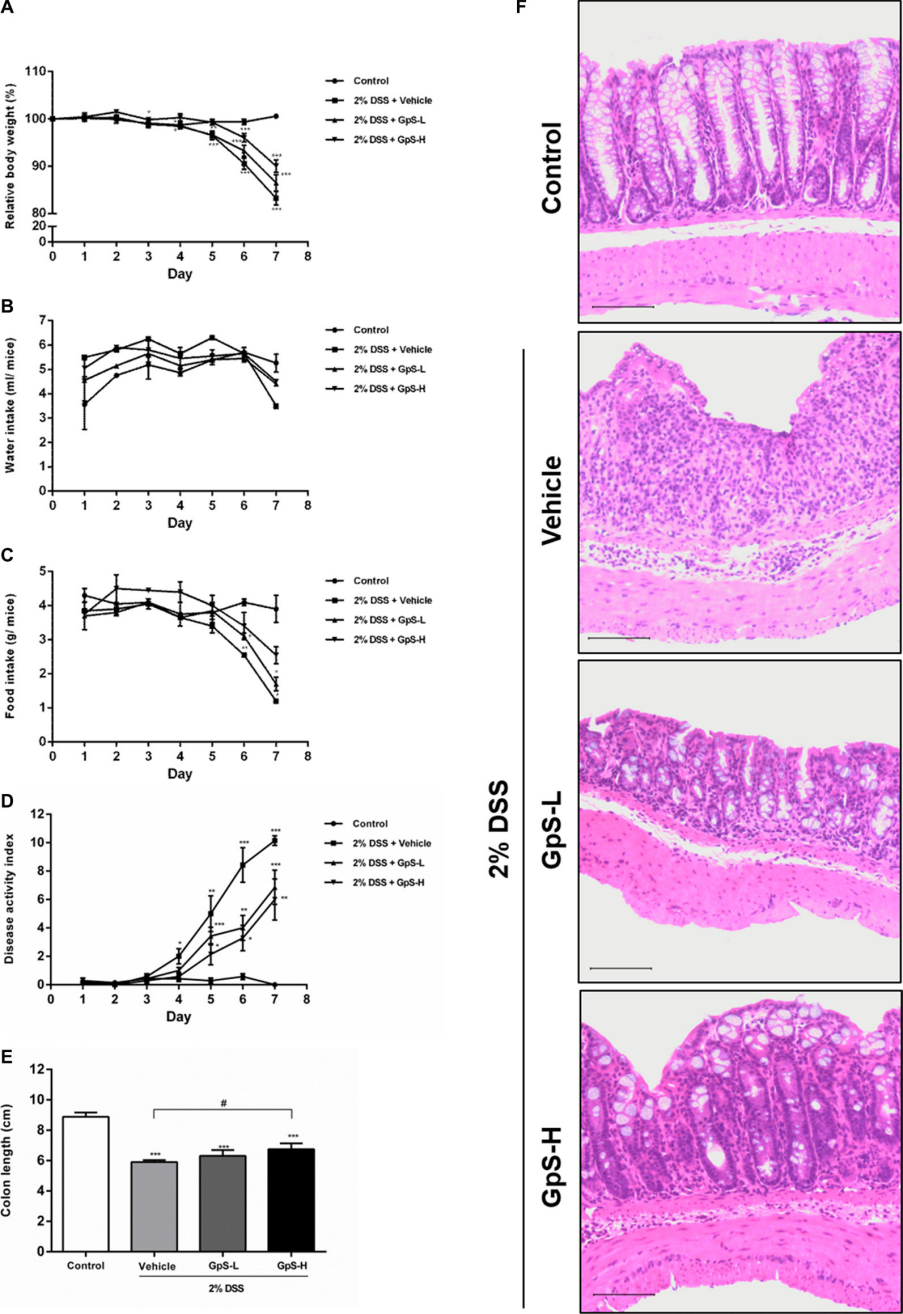
GpS ameliorates the severity of DSS-induced acute colitis in mice Acute colitis was induced by supplying mice with 2% DSS in their drinking water for seven consecutive days. Mice were orally administered with vehicle (0.5% CMC), GpS-L (500 mg/kg GpS in 0.5% CMC) or GpS-H (800 mg/kg GpS in 0.5% CMC) alongside DSS induction. Control mice received filtered water and 0.5% CMC. (**A**) Body weights were measured daily (*n* = 7 per group) and are presented as the group mean percentage change from initial ± SEM. Daily changes in (**B**) water consumption, (**C**) food intake, (**D**) disease activity index were measured. At the experimental endpoint, (E) colon lengths were measured. (**F**) Histological changes were visualized by hematoxylin and eosin staining of paraffin embedded colon sections and assessed under light microscopy. Control group shows normal colon histology of mice. Vehicle group shows typical acute colitis features, including destruction of epithelial integrity and crypts and inflammatory cell infiltration in the colons. GpS treatment group retains the epithelial cell architecture and reduces the morphological alteration resulting from DSS treatment. Data are expressed as means ± SEM; ^*^P < 0.05, ^**^P < 0.01, ^***^P < 0.001 compared to uninduced control; ^#^*P* < 0.05, ^##^*P* < 0.01, ^###^*P* < 0.001 compared to 2% DSS-induced vehicle group.

The DSS-induced acute colitis mouse model is a well-established animal model for the study of IBD [33]. Using this model, we examined the anti-inflammatory and therapeutic effects of GpS on IBD *in vivo*. As four weeks of treatment with 500 or 750 mg/kg GpS in *Apc^Min/+^* mice did not induce significant adverse effects in our previous study [32], we selected 500 mg/kg (low dose; GpS-L) and 800 mg/kg (high dose; GpS-H) doses of GpS for this study. Administration of 2% DSS in drinking water for 7 consecutive days successfully induced weight loss, and decreased food and water intake in mice treated with vehicle (Figure 4A–4C). In comparison, treatment with GpS-L or GpS-H during DSS administration rescued weight loss and improved food and water intake, as well as decreased DSS-induced disease activity index (DAI) values (Figure 4A–4D). Moreover, GpS treatment also ameliorated colon shortening in mice, from a 25% reduction to 21% and 15% with GpS-L or GpS-H treatment respectively (Figure 4E). Histological analysis also indicated the beneficial effects of GpS treatment. As shown in Figure 4F, colon sections from DSS-induced mice under vehicle treatment exhibited the inflammatory cell infiltration and destruction of epithelial cell architecture typical of acute colitis, whereas treatment with GpS-L or GpS-H ameliorated the damage induced by DSS. In addition, colon sections from mice receiving GpS-H treatment showed minimal epithelial damage or crypt loss when compared with vehicle control, and strikingly, these results were comparable to the uninduced control group.

### GpS suppressed cytokine production in the colons of DSS-induced acute colitis mice

**Figure 5:**
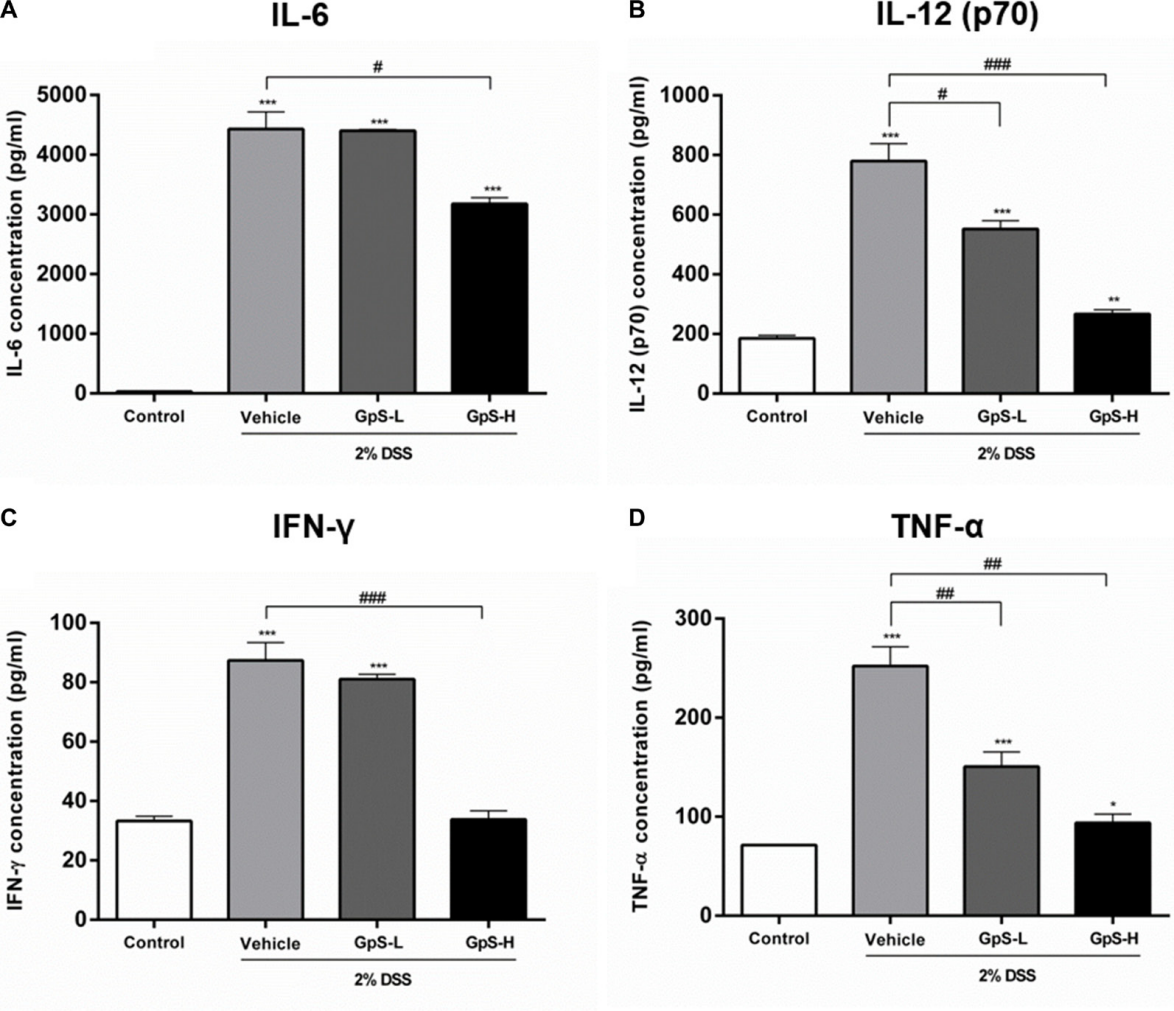
GpS suppresses proinflammatory cytokine production of colons from mice with DSS-induced acute colitis Colon sections from mice were removed at the end of experiment, cultured and the conditioned media pooled for Bioplex mouse cytokine assay. Measurement of (**A**) IL-6, (**B**) IL-12 (p70), (**C**) IFN-γ and (**D**) TNF-α in colon culture media. Data are expressed as means ± SEM; ^*^P < 0.05, ^**^P < 0.01, ^***^P < 0.001 compared to uninduced control; ^#^*P* < 0.05, ^##^*P* < 0.01, ^###^*P* < 0.001 compared to 2% DSS-induced vehicle group.

**Figure 6:**
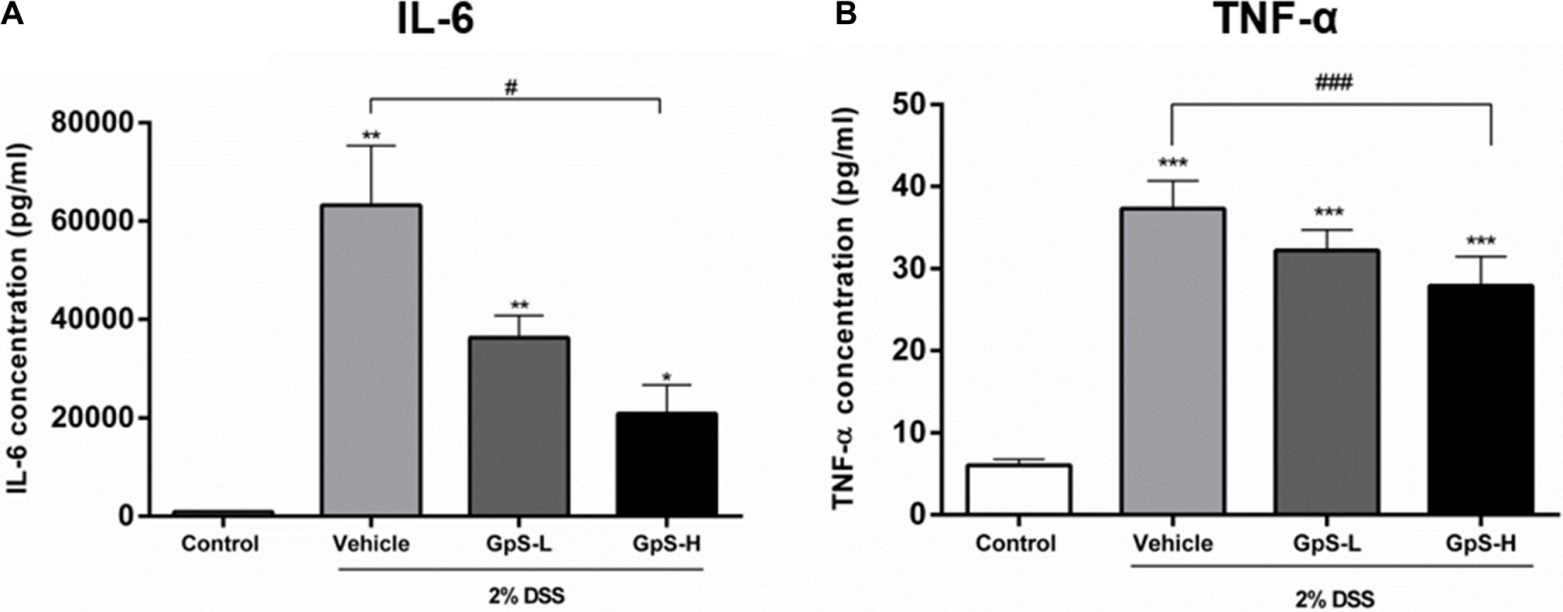
Validation of the suppressive effect of GpS on cytokine production in mice colons with DSS-induced acute colitis Concentrations of (**A**) IL-6 and (**B**) TNF-α in the colon cultures of individual mice from each group (*n* = 3) were measured by ELISA. Data are expressed as means ± SEM; ^*^P < 0.05, ^**^P < 0.01, ^***^P < 0.001 compared to uninduced control; ^#^*P* < 0.05, ^##^*P* < 0.01, ^###^*P* < 0.001 compared to 2% DSS-induced vehicle group.

A dysregulated immune system is known to be one of the major contributors to the development of IBD, leading to a proinflammatory cytokine profile in IBD patients [3, 4, 34]. To examine the effect of GpS on cytokine profiles in mice, we used organ primary culture, which has previously been used to study cytokine production in intestinal disorders [33, 35]. Colon sections obtained from mice after sacrifice were cultured, and the conditioned media pooled and analyzed using a Bio-Plex Pro Mouse Cytokine23-Plex Panel kit. As shown in Figure 5, production of IL-6, IL-12 (p70), IFN-γ and TNF-α were significantly increased in colons of DSS-induced mice when compared with uninduced control. In comparison with vehicle treatment, GpS-L or GpS-H significantly downregulated production of these proinflammatory cytokines in DSS-induced mice (Figure 5). As IL-6 and TNF-α are two of the most dysregulated cytokines in IBD patients [36], we further validated their levels in the conditioned media of individual mice colons. In agreement with the results from the Bioplex cytokine assay, GpS-L or GpS-H significantly downregulated the production of IL-6 and TNF-α in mice colons when compared to vehicle (Figure 6).

### GpS suppressed NF-κB and STAT3 signaling in colons of DSS-induced acute colitis mice

**Figure 7:**
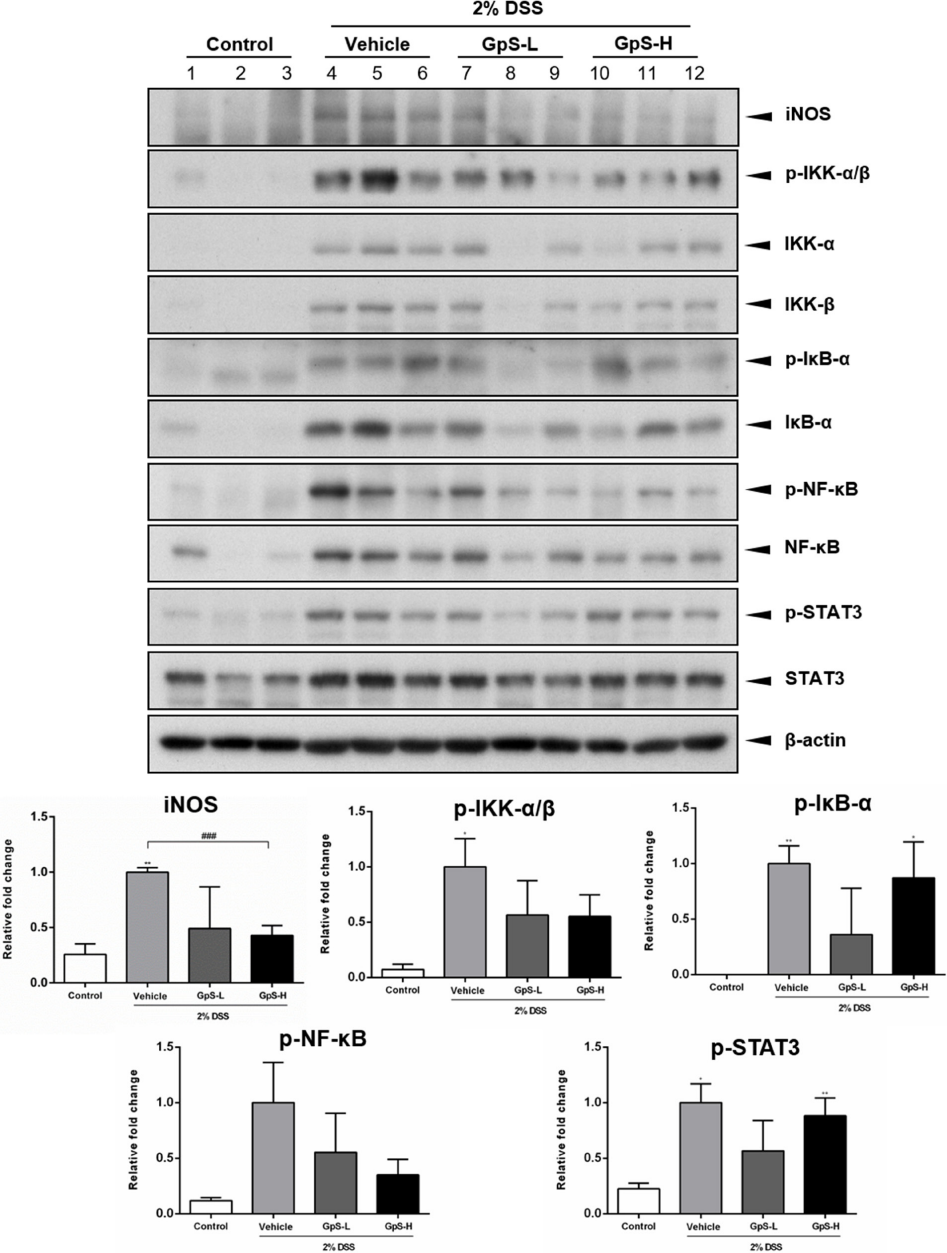
GpS suppresses iNOS, NF-κB and STAT3 signaling in colonic tissue of DSS-induced mice Colon tissues were lyzed and analyzed by Western blotting for iNOS, phospho-IKK-α/β, IKK-α, IKK-β, phospho-IκB-α, IκB-α, phospho-NF-κB, NF-κB, phospho-STAT3 and STAT3. β-actin was used as an internal loading control. Representative immunoblots and their quantifications are shown. Data are expressed as means ± SEM; ^*^P < 0.05, ^**^P < 0.01, ^***^P < 0.001 compared to uninduced control; ^#^*P* < 0.05, ^##^*P* < 0.01, ^###^*P* < 0.001 compared to 2% DSS-induced vehicle group.

**Figure 8:**
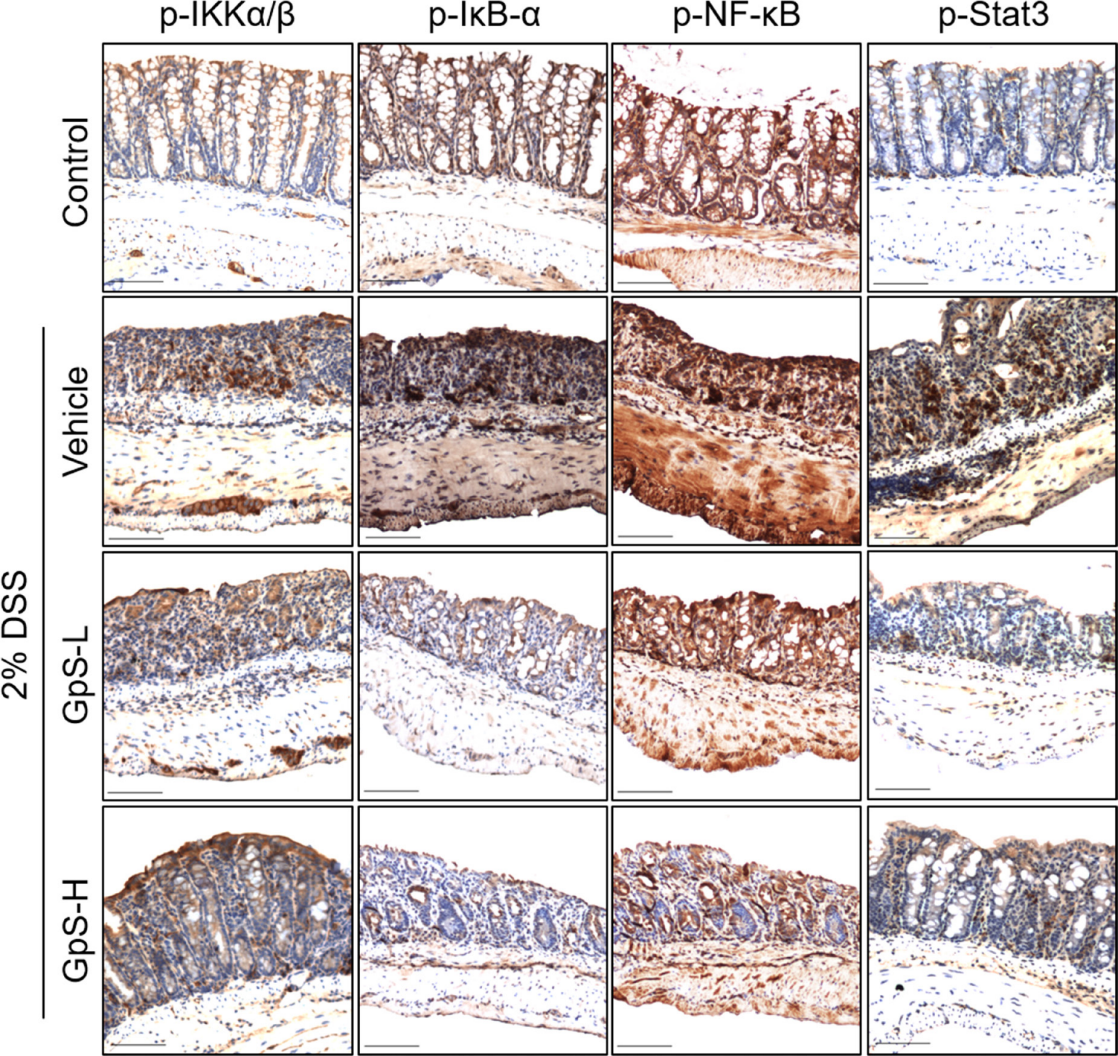
GpS inhibits the activation of NF-κB and STAT3 signaling in DSS-induced mice Paraffin embedded colon sections were analyzed by immunohistochemical staining for phospho-IKK-α/β, phospho-IκB-α, phospho-NF-κB and phospho-STAT3.

Based on the above results, we further examined the effects of GpS on iNOS, NF-κB and STAT3 signaling in the colons of DSS-induced mice. As shown in Figure 7, GpS treatment dose-dependently downregulated the DSS-induced expression of iNOS when compared with vehicle. In addition, GpS also mediated the expression of phosphorylated IKK-α/β, IκB-α, NF-κB and STAT3, echoing the results from our *in vitro* experiments. To confirm these results, we examined the expression of these four proteins in mice colons using immunohistochemical (IHC) analysis. In accordance with the Western blot results, GpS-L or GpS-H treatment reduced the DSS-induced expression of phosphorylated IKK-α/β, IκB-α, NF-κB in mice colons (Figure 8).

## DISCUSSION

As a chronic, relapsing gastrointestinal inflammatory disorder, the treatment goals for IBD are to induce and maintain remission of symptoms and mucosal inflammation, ultimately improving the quality of life of the IBD patient [37]. At present, 5-ASA and corticosteroids remain as the major treatment options for IBD. However, side effects and adverse events from these treatments can hinder their therapeutic selection [38, 39]. In the past several years, a host of new treatment options have been developed or are currently in clinical trials, including immunotherapies such as anti-TNF or anti-IL-12/23 antibodies. Although these agents have been shown to be quite effective and safe, they do not treat the underlying cause of the disease and are only effective in a subset of the population [40, 41]. As the incidence and prevalence of IBD continues to rise worldwide [1, 2], demand for potent, non-toxic and effective therapeutics for treatment of the disease is increasing. *Gynostemma pentaphyllum* has been widely used as a herbal medicine in Asian countries, and its saponins (GpS) have been shown to possess potent anti-inflammatory effects. Therefore, we have performed a systematic study to investigate the anti-inflammatory effect of GpS and its underlying mechanisms in IBD.

It has been shown that overexpression of iNOS and the subsequent production of NO are associated with the initiation and maintenance of IBD. In the active state of IBD, NO concentration and iNOS protein expression were increased in colons of patients when compared with control, suggesting a potential role of NO and iNOS in mediating the pathogenesis of IBD [7, 8, 42]. As in previous studies [31], our current study identified a strong suppressive effect of GpS on iNOS protein expression and NO production in LPS-induced macrophages, indicating the potential efficacy of GpS in IBD.

NF-κB has been shown to mediate the activation of iNOS as an upstream regulator [43]. In the presence of stimuli such as LPS or bacteria, the IKK-α/β complex is phosphorylated, resulting in phosphorylation of NF-κB-bound IκB. Consequently, phosphorylated IκB is targeted for proteosomal degradation and NF-κB is released. Nuclear translocation of NF-κB then increases the transcription of genes encoding proinflammatory molecules including chemokines, cytokines and proinflammatory enzymes, forming an amplifying feed-forward loop [11, 13, 44]. It has been shown that NF-κB is markedly induced in the intestines of IBD patients and its expression level strongly influences progression of the disease [10, 11], therefore suppression of NF-κB activation is a major focus of IBD treatment. In addition, emerging evidence has suggested a role for STAT3 in IBD. It has been shown that STAT3 signaling is constitutively activated in IBD patients and that activation of STAT3 is responsible for triggering the innate immune response, leading to the production of proinflammatory mediators [12]. Thus, we focused on the effect of GpS on the NF-κB and STAT3 signaling pathways. Our results clearly showed that GpS treatment could suppress the nuclear translocation of NF-κB/p65 in LPS-induced macrophages. In addition, when compared with vehicle control, GpS significantly inhibited the protein expression of phospho-NF-κB and phospho-STAT3 in a time and dose dependent manner in the macrophages, suggesting the anti-inflammatory potential of GpS.

We furthered our study in a chemically-induced colitis mouse model to examine the *in vivo* anti-inflammatory effect of GpS. The DSS-induced acute colitis mouse model is a well-established animal model for the study of IBD which mimics the disease manifestation and histopathological characteristics of IBD patients, making it advantageous to other animal models for *in vivo* studies [45]. Mice under vehicle treatment exhibited typical acute colitis manifestations, including weight loss, shortening of colon length and increased DAI. Microscopic evaluation of mice colons showed that GpS treatment improved the DSS-induced inflammatory cell infiltration and destruction of epithelial cell architecture.

As proinflammatory cytokines are responsible for mucosal injury and consequential tissue damage in IBD patients via the induction of proinflammatory signaling pathways [36, 46], suppression of proinflammatory cytokine production and signaling pathways would be beneficial for treatment of disease. Using organ primary culture and cytokine assays, we examined the effect of GpS on the cytokine production of the colons of DSS-induced mice. Our results demonstrated that GpS treatment attenuated the production of proinflammatory cytokines, which may be beneficial for inhibiting the progression of IBD [47]. More importantly, the production of TNF-α and IL-6 were significantly suppressed by GpS treatment. It has been shown that in addition to being target genes of activated NF-κB and STAT3 signaling, TNF-α and IL-6 could also trigger the activation of NF-κB and STAT3 signaling [12, 48, 49]. As colon biopsies and sera from IBD patients have increased concentrations of TNF-α and IL-6, the suppressive effect of GpS on TNF-α and IL-6 production in the colons of colitis mice may also indicate the potential inhibitory effect of GpS on NF-κB and STAT3 signaling. As expected, GpS treatment suppressed the proteins involved in NF-κB and STAT3 signaling. More importantly, we found that GpS potently downregulated phospho-NF-κB in both cell and animal experiments, suggesting that GpS may act as an inhibitor of NF-κB in the treatment of IBD, suppressing the production of proinflammtory cytokines and consequential tissue damage in patients.

**Figure 9:**
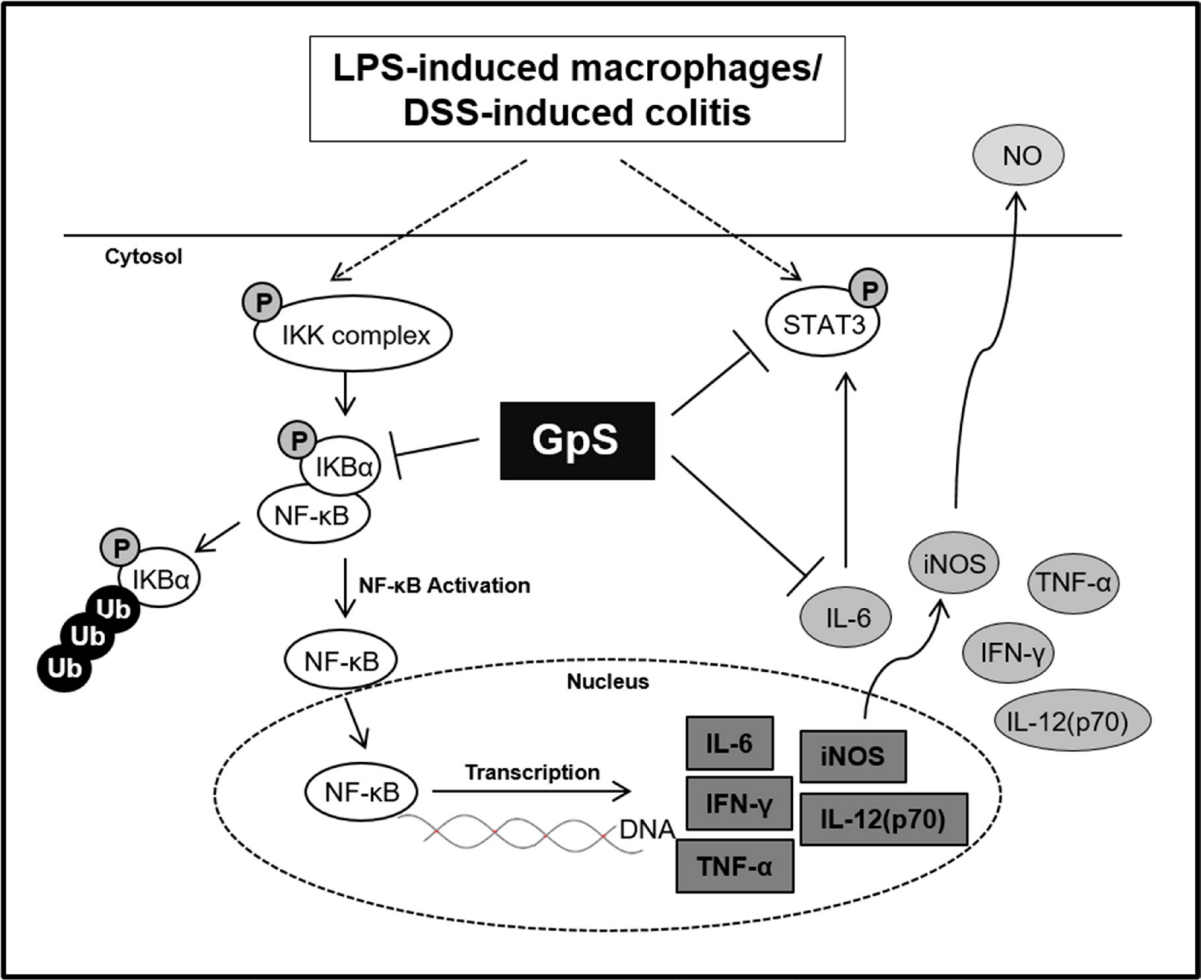
A schematic diagram proposing the potential inhibitory role of GpS in inflammatory signaling pathways GpS potentially attenuated inflammatory responses through inhibiting the activity of NF-κB and STAT3 in LPS-induced RAW264.7 macrophages and DSS-induced acute colitis mice.

In conclusion, we have provided evidence of the anti-inflammatory effects of GpS *in vitro* and *in vivo*. GpS could inhibit the production of proinflammatory cytokines and suppress activation of the proinflammatory NF-κB and STAT3 signaling pathways in colons of DSS-induced acute colitis mice, while improving their condition (Figure 9). Our data not only provides evidence of the anti-inflammatory effects of GpS, but also highlights the potential of GpS as a therapeutic agent for the treatment of IBD.

## MATERIALS AND METHODS

### Preparation of GpS

GpS were purchased from Hauduo Natural Products (Guangzhou, China). Authentication and chemical profiling of each batch was conducted in our laboratory as previously reported [32].

### Cell culture and *in vitro* stimulation of macrophages

RAW264.7 murine macrophages were maintained as previously described [50]. For the cell viability assay, 1 × 10^4^ RAW264.7 macrophages were plated in a 96-well plate for 24 hrs and then treated with GpS at concentrations ranging from 3.1–200 μg/ml for 24 hrs or treated with GpS (3.1–200 μg/ml) for 1 hr before co-treatment with 1 μg/ml LPS (Sigma-Aldrich, St. Louis, MO, USA) for 24 hrs. Cell viability was then measured using MTT (Sigma-Aldrich). For NO and cytokine production measurement and Western blotting, 1 × 10^6^ RAW264.7 macrophages were treated with 0, 100, 150, or 200 μg/ml GpS 1 hr before co-treatment with LPS (1 μg/ml) and GpS for 24 hrs or as indicated. Cells without treatment served as controls. After the treatment period, culture medium was collected and centrifuged to remove debris. Media was then aliquoted and stored at −80°C until use. Cells were washed with PBS and cell pellets collected were stored at −80°C until use. For immunofluorescence staining of NF-κB/p65, procedures were carried out following those by *Qian et al*. [51], with slight modifications. Briefly, 1 × 10^6^ RAW264.7 macrophages were seeded in a μ-Slide 8 well chambered coverslip (ibidi, Martinsried, Germany) for 24 hrs. Cells were pretreated with or without GpS (200 μg/ml) for 12 hrs and then stimulated with LPS (1 μg/ml) for 1 hr. Cells were then fixed, permeablized and stained for NF-κB. Nuclei were stained with 300 nM DAPI solution (Molecular Probes, Life Technologies) and then mounted with fluorescent mounting medium (Dako, Agilent, Santa Clara, CA, USA). Cells were examined by fluorescence microscopy using a Leica DMI 3000B inverted microscope (Leica Microsystems, Wetzlar, Germany).

### Measurement of NO production

NO levels in conditioned media from RAW264.7 macrophages were determined using the Griess Reagent System according to the manufacturer's instructions (Promega, Madison, WI, USA).

### Animals and DSS-induced acute colitis

Wild-type C57BL/6J mice were bred and acute colitis was induced as described previously [50]. Animals were randomly assigned into 3 groups (*n* = 7 per group) and were induced by providing 2% w/v DSS (reagent grade; 36 000–50 000 Da; MP Biomedicals, Solon, OH, USA) in drinking water for seven consecutive days. Mice were treated daily by gavage as follows: (a) vehicle: 0.5% CMC; (b) low dose GpS (GpS-L): 500 mg/kg GpS in 0.5% CMC and (c) high dose GpS (GpS-H): 800 mg/kg GpS in 0.5% CMC. During the treatment period, mice continued to receive 2% DSS in their drinking water. A control group (*n* = 7) received drinking water (without DSS) with vehicle treatment (0.5% CMC). All animal experiments were approved by the Animal Subjects Ethics Sub-Committee (ASESC) of The Hong Kong Polytechnic University and conducted in accordance with the Institutional Guidelines and Animal Ordinance of the Department of Health, Hong Kong S.A.R..

### Assessment of colitis

Body weight, food and water consumption were measured daily throughout the experiment. Stool consistency and visible blood in feces were also examined. DAI was determined according to criteria reported by *Marín et al*. [52], parameters of which are outlined in Table 1. At the time of sacrifice, intestines were removed and colon lengths were measured. Colons were then opened longitudinally and washed with saline. Colon sections were either fixed in formalin solution for histopathological assessment, cultured in medium for cytokine level assessment or snap frozen at −80°C for protein isolation.

**Table 1:** Disease activity index (DAI^*^) scoring system parameters

Score	Weight loss	Stool consistency	Visible blood in feces
0	None	Normal	None
1	1–5%		
2	6–10%	Loose	Slight bleeding
3	11–20%		
4	< 20%	Diarrhea	Gross bleeding

^*^DAI value is calculated as the sum of scores of weight loss, stool consistency and visible blood in feces.

### Colon tissue culture

Colon tissue culture was performed as described by *Wirtz et al*. [33] with slight modifications. Briefly, isolated mice colon sections approximately 1 cm in length were washed thoroughly with sterile PBS and cultured in RMPI 1640 culture medium supplemented with 10% FBS at 37°C for 24 hrs. Conditioned media was centrifuged to remove debris and the supernatant was collected. Cytokine production was assessed using Bioplex mouse cytokine assay and ELISA.

### Bioplex mouse cytokine assay and measurement of cytokine production

Cytokine levels of the colon tissue cultures were measured using the Bio-Plex 200 System (Bio-Rad, Hercules, CA, USA) with the Bio-Plex Pro Mouse Cytokine23-Plex Panel kit (Bio-Rad), according to the manufacturer's instructions. Concentration of IL-6 and TNF-α were measured using ELISA kits (BioLegend, Cambridge, UK) according to the manufacturer's instructions.

### Western blot analysis

RAW264.7 cell pellets and colon tissues were lysed in RIPA buffer (50 mM Tris-HCl, pH 7.4, 150 mM NaCl, 1 mM EDTA, 1% Triton X-100, 1% sodium deoxycholate, 0.1% SDS) and then centrifuged to remove debris. Western blotting was carried out as previously reported [50] using the following antibodies: β-actin (Santa Cruz Biotechnology, Santa Cruz, CA, USA), iNOS (BD Biosciences, CA, USA), IKK-α, IKK-β, phospho-IKK-α/β, IκB-α, phospho-IκB-α, NF-κB, phospho-NF-κB, STAT3 and phospho-STAT3 (Cell Signaling Technology, Danvers, MA, USA).

### Immunohistochemical staining

Expression of phospho-IKK-α/β, phospho-IκB-α, phospho-NF-κB and phospho-STAT3 in colon tissues were evaluated using IHC staining as previously reported [32].

### Statistical analysis

Statistical analyses of cell viability, NO and cytokine production, mice body weight, food and water consumption, colon length and immunoblot densitometry were conducted using a one way analysis of variance (ANOVA). ^*^P < 0.05, ^**^P < 0.01 and ^***^P < 0.001 were considered as significant differences. Data are presented as mean ± SD (cell experiments) or mean ± SEM (animal experiments) of three independent experiments.

## Abbreviations

IBD: Inflammatory bowel disease
GpS: *Gynostemma pentaphyllum* saponins
UC: Ulcerative colitis
CD: Crohn's disease
iNOS: Induced nitric oxide synthase
COX-2: Cyclooxygenase-2
IL: Interleukin
TNF-α: Tumor necrosis factor-alpha
CRC: Colorectal cancer
ROS: Reactive oxygen species
LPS: Lipopolysaccharide
DSS: Dextran sulfate sodium (DSS)
DAI: Disease activity index
IHC: Immunohistochemical
ANOVA: Analysis of variance.
